# Improving filter recommendations: the role of individual light sensitivity in ecological conditions

**DOI:** 10.3389/fpsyg.2026.1755789

**Published:** 2026-06-30

**Authors:** Sarah Marié, Chloé Moger, Romain Brasselet

**Affiliations:** 1 Research & Foresight, Global Lens Innovation, EssilorLuxottica, Paris, France; 2 Sorbonne Université, Institut National de la Santé et de la Recherche Médicale, Centre National de la Recherche Scientifique, Institut de la Vision, Paris, France

**Keywords:** discomfort glare, filters, light sensitivity, model, outdoor

## Abstract

**Introduction:**

This study investigates the optimal transmittance settings for discomfort glare filters under ecological outdoor conditions, accounting for individual light sensitivity and varying light conditions. The objective is to guide the personalization of sunglass transmittance to enhance visual comfort in various outdoor environments.

**Methods:**

Twenty-eight participants (18 women and 10 men, aged 18–61) were equipped with electrochromic glasses with a visible light transmittance ranging from 80% to 5%. For each participant, preferred transmittance was assessed in three light conditions (facing the sun, back to the sun, and in the shade) using three methods (clearest-to-dark, darkest-to-clear, and manual settings). Individual light sensitivity was quantified using the Eye Resistance Score (ERS) measured by the Lumiz 100™ device. A logistic model was designed to predict the optimal transmittance settings.

**Results:**

A total of 1,530 transmittance settings were recorded. Illuminance, method, and light sensitivity (ERS) all significantly contributed to the model predicting optimal transmittance. Less sensitive participants required category 2 filters (43–18% transmittance) for high illuminations, while more sensitive participants needed category 4 filters (8–3% transmittance) under equivalent light conditions for direct sunlight.

**Conclusion:**

Personalized filter recommendations based on individual light sensitivity and outdoor conditions can enhance visual comfort. This study highlights the importance of considering individual variability in light sensitivity to optimize discomfort glare protection. Further research in diverse environments and lighting conditions could help refine these recommendations.

## 1 Introduction

Discomfort glare is a common phenomenon encountered in everyday life, whether outdoors on a sunny day, while driving at night and facing oncoming headlights, or indoors under varying lighting conditions. Discomfort glare refers to “glare that causes annoyance” (Mainster and Turner, 2012). The retina acts as a conduit, transmitting information about illumination levels that are either too intense or excessively variable in a given context. Pierson et al. (2018) further distinguish between two types of discomfort glare:

- *Saturation or absolute glare*: This occurs when the total amount of light reaching the observer’s eyes is excessively high, such as intense sunlight experienced on a bright day.
- *Contrast or relative glare*: Here discomfort arises from the contrast between a bright spot and the background. An example is the glare encountered while driving at night, particularly when facing oncoming headlights.

*Exploring discomfort glare*: Most investigations into discomfort glare have been conducted by lighting engineers, primarily focusing on contrast glare conditions (Bennett, 2013; Hopkinson, 1940; Iwata et al., 1992; Luckiesh and Holladay, 1925; Saur, 1969). Predictive models have been developed based on subjective ratings, considering parameters such as apparent size, localization, spectrum, intensity of light sources, and background levels. These models are valuable tools for designing indoor lighting installations.

However, a critical gap remains: these models often overlook interindividual differences. Despite studies highlighting such variations (Hopkinson, 1940; Osterhaus, 2001; Rodriguez et al., 2016; Saur, 1969), they have not been fully integrated into the existing models. Yun et al. (2011) conducted a comparative study between two groups of subjects: “glare-sensitive” and “glare-insensitive,” emphasizing the need to account for individual variability.

*Physiological factors and glare sensitivity*: Researchers have explored physiological factors associated with glare sensitivity (reviewed by Pierson et al., 2018). Notably:

- *Gender and age*: Glare ratings appear largely unaffected by gender (Iwata et al., 1992; Jia, 2014; Kent et al., 2016; Saur, 1969; Vanagaite et al., 1997; Verriotto et al., 2017) or age (Jia, 2014; Marié et al., 2021; Saur, 1969; Van Den Wymelenberg, 2012) in most studies.
- *Pupil parameters*: Pupil size and fluctuations were investigated as potential indicators of discomfort. However, the correlations between pupil parameters and discomfort ratings remained moderate (*r*^2^ < 0.5) (Lin et al., 2015; Stringham et al., 2011; Tyukhova and Waters, 2019).
- *Self-evaluation challenges*: Self-assessment of glare sensitivity poses difficulties, warranting further exploration. Compared to the discomfort threshold measurement, the correlation is low (Kent et al., 2016; Marié et al., 2021; Van Den Wymelenberg, 2012).

The Lumiz 100™ (Essilor International, Paris, France) has been developed (Montés-Micó et al., 2020) to measure light sensitivity that can in turn be used to provide filter recommendations.

*Solution for outdoor discomfort glare*: Sunglasses are widely recognized as an effective solution for protecting the eyes from solar ultraviolet radiation and reducing the risk of eye damage and cancer (Gies et al., 2018; Izadi et al., 2018; Maddock et al., 2009; Majdi et al., 2014). Recent studies have explored the social dynamics associated with wearing sunglasses (Graham and Ritchie, 2019; Takehara et al., 2023). In sports activities, studies have been conducted on the preferences of polarizing filters (Mercatelli, 2023) and tint colors (Cerviño et al., 2008; Christie et al., 2023; Erickson et al., 2009). However, the effect of transmittance level on discomfort glare remains underexplored.

In this study, we revisited the topic of discomfort glare filters, focusing on their transmittance properties. Our investigation draws from historical studies, including Luria’s work which evaluated six neutral density filters across age groups in two seasons (summer and winter) (Luria, 1984). Observers preferred sunglasses that reduced light levels to 1,000–1,400 cd/m^2^, indicating a need for higher filter densities than those typically found in commercial products. Most participants chose filters with 8.9% or 2.5% transmittance. The presence of individual variation, with a notable minority favoring filters outside this range, highlights the importance of offering a broader selection of filtering options.

Norms ISO 12312-1 on Eye and face protection - Sunglasses and related eyewear - Part 1: sunglasses for general use have been established to harmonize labeling, ensure driving safety (lenses with transmittance below 8% are unsuitable for driving) (Dain, 2003), and provide UV protection (Pitts, 1993). The transmission of filters can be reported as Optical Density (OD) or Transmittance (Tr). OD is a logarithmic measure of light attenuation, defined as log₁₀(1/Tr), where Tr is luminous transmittance. OD is particularly suited for modeling visual responses across wide illumination ranges (further used in this paper), while luminous transmittance (%) remains more intuitive for clinical interpretation and regulatory classification. The categories of lens tint in sunglasses are regulated based on the amount of visible light, UV and IR transmitted (Only visible light reported here). The analysis conducted in this study relies on ISO 12312-1, ISO8980-3, and AS1067 description of category. Category 0 lenses have a very light tint with luminous transmittance greater than 80% (OD < 0.10), making them suitable for overcast or indoor environments and the only category approved for night driving. Category 1 lenses feature a light tint with transmittance greater than 43% and up to 80% (0.10 ≤ OD < 0.37), recommended for low sunlight conditions. Category 2 lenses, with medium tint and transmittance greater than 18% and up to 43% (0.37 ≤ OD < 0.74), are appropriate for average sunlight. Category 3 lenses offer a dark tint with transmittance greater than 8% and up to 18% (0.74 ≤ OD < 1.10), ideal for bright sunlight. Finally, Category 4 lenses have a very dark tint with transmittance greater than 3% and up to 8% (1.10 ≤ OD < 1.52), designed for extremely bright environments such as high-altitude mountaineering, but are prohibited for driving during daylight because their high attenuation can lead to insufficient visibility.

Building on the existing research, we propose to adapt filter darkness to individual sensitivity in environments with different outdoor light conditions. Despite the prevalence of category 3 filters on the market, personalized filter density adjustments could enhance overall satisfaction with sunglasses.

To conduct our study, we used a novel solution: electrochromic glasses. These lenses developed internally at Essilor enable real-time transmittance adjustments via ambient light sensors or smartphone commands by modulating the electric field applied to the electrochromic material. This allows the transmittance to be modified without requiring the participants to remove glasses and be exposed directly to the current light conditions. Transmittance can be set from 0.1 to 1.2 OD in steps of 0.1 OD, with each step change occurring in less than 1 s. Filters were calibrated to ensure reliable transmittance values. By developing psychometric methods, we aimed to determine the optimal protection from discomfort glare (visible light transmittance) for different individuals based on outdoor light conditions. For the first time, transmittance settings were measured in real outdoor conditions using psychometric methods, without subjective bias that can be produced by the observation of transmittance level when participants changed glasses.

## 2 Materials and methods

This study was reviewed and approved by an independent ethical review board (CPP Ile de France VII reference number ANSM RCB: 2021-A01249-32) and conformed to the principles and applicable guidelines for the protection of human subjects in biomedical research.

Twenty-eight participants participated in a study conducted over one to four visits in summer (from June to September) between 12 p.m. and 4 p.m. The primary objective was to assess transmittance filter preferences using electrochromic glasses outdoors under 3 light conditions using 3 methodologies in a fully randomized order. A total of 85 visits were conducted. The experiment was conducted in an urban environment, specifically in Paris, France (Latitude: 48.85332, Longitude: 2.34885), with surrounding buildings.

### 2.1 Experimental setup

- Participants: Twenty-eight individuals participated in the study.
- Electrochromic Glasses: The glasses had a gray tint and offered a visible light transmittance range of 80 to 5%. An integrated smartphone application allows the examiner to adjust the transmittance settings and record the illumination data. The application interfaces with a calibrated ambient light sensor that was strategically positioned at the front of the frame to measure vertical illuminance.
- Light Conditions: Three light conditions were assessed during each visit:

1. Facing the Sun: Participants stood in direct sunlight and were asked to look at sunny objects or areas (excluding direct sun exposure).
2. Back to the Sun: Participants faced away from the sun while still in the sunlight and were asked to observe sunny objects or areas.
3. In the Shade: Participants were in shadowed areas of trees, and were asked to look at objects, or areas within the shade.

Methodologies Explored:

1. *Clearest-to-dark method*: Starting at maximum filter transmittance (80%), the examiner gradually decreased the transmittance using a smartphone application until the participants felt comfortable and no longer disturbed by light. The instruction was: “I’ll gradually increase the tint level of the lenses, and you’ll let me know when you feel comfortable, when you are no longer bothered or dazzled by the light or the sun.”
2. *Darkest-to-clear method*: Starting at minimum filter transmittance (5%), the examiner manually increased the transmittance level until the participants reported discomfort due to light. The results of the previous step were recorded. The instruction was: “I’m going to gradually decrease the tint level of the lenses. Please let me know as soon as you start feeling uncomfortable, bothered, or dazzled by the light.”
3. *Manual setting method*: Participants manually adjusted the filter transmittance, starting at 50%, using a remote with up and down buttons allowing step changes (0.1 OD). They were only asked to adjust the filter transmittance to be comfortable under current light conditions. No indication of transmittance level was provided. The instruction was: “Please use the remote control up and down buttons to adjust the tint level of the lenses yourself and choose the setting that feels most comfortable in the current lighting conditions. The lenses are slightly tinted to start with, and you can either increase or decrease the tint as needed.”

*Data collection*: A total of 1,530 transmittance settings were recorded, with up to 72 settings per participant. The order of the conditions and methods was randomized for each visit.

*Eye resistance score*: Before outside measurements, the participants’ light sensitivity was evaluated indoors using the Lumiz 100™ device, a portable, wireless device used to clinically assess light discomfort thresholds. It features a uniformly illuminated spherical white surface to avoid direct light exposure to LEDs. The device uses two LED sources, warm (4,000°K) and cold (6,500°K), to simulate natural and artificial lighting. Users indicate two levels of discomfort: *just perceptible* and *really disturbing*. Measurements are taken under three lighting conditions: gradually increasing warm light, gradually increasing cold light, and flashing warm light. These scenarios reflect everyday lighting environments, with continuous lighting allowing adaptation and flashing light testing rapid visual response. This tool assigns an Eye Resistance Score (ERS). A complete description of the measurements is available in Montés-Micó et al. (2020).

### 2.2 Modeling

The transmittance of ophthalmic filters ranges from 3% to 100% (optical density from 0 to 1.52). From a purely theoretical standpoint, this naturally calls for using a nonlinear model that is lower-bounded by 0. We also observed some saturation, as detailed in the results section. We therefore opted for a lower- and upper-bounded function to model the transmittance as a function of illuminance (both in decimal logarithm), and in this specific case, we opted for a logistic function.

#### 2.2.1 Initial model using only illuminance

A very simple first model of Transmittance *T* (in OD) as a function of Illuminance *I* (that disregards participant, method and ERS, henceforth referred to as model 0) was defined:


$$\text{model}\;\;0:T=d+\frac{a}{1+e^{-b.(I+c)}}$$

This is a simple logistic curve defined by its amplitude *a*, its slope *b*, its baseline horizontal shift *c* and its lower asymptote *d*. It is implemented in R as stats::nls(Transmittance ~ d + a*(1/(1 + exp(−b*(Illuminance + c)))).

Note that for later comparisons, and to account for repeated measures, we also implemented a similar model (model 1) that includes participant ID as a random effect inside the argument of the logistic function so that each participant can have their own baseline illuminance at which they need a transmittance of 50%: nlme::nlme(Transmittance ~ d + a*(1/(1 + exp(−b*(Illuminance + c)))), fixed = a + b + c + d ~ 1, random = c ~ 1 | ID).

#### 2.2.2 Advanced models using illuminance, method and/or eye resistance score

Since we hypothesized that the transmittance *T* settings (in OD) would differ according to the method and the light sensitivity of participants (ERS), more models (model 2, 3 and 4) including the effect of Method and/or ERS were developed using the following equations:


$$\text{model}\;\;2:T=d+\frac{a}{1+e^{-b.(I+c+e.\mathrm{ERS})}}$$

$$\text{model}\;\;3:T=d+\frac{a}{1+e^{-b.(I+c+\text{Method})}}$$

$$\text{model}\;\;4:T=d+\frac{a}{1+e^{-b.(I+c+\text{Method}+e.\mathrm{ERS})}}$$

These are logistic curves defined by an amplitude *a*, a slope *b*, a baseline horizontal shift *c*, a lower asymptote *d*, the effect of the continuous ERS *e*, and the differential effects of the discrete methods. These models assume that the effect of ERS and Method is to shift the logistic function horizontally without altering its shape. In addition, every subject may have their own horizontal shift mathematically implemented as a random effect. This is typically implemented in R as: nlme::nlme(model = Transmittance ~ d + a * (1/(1 + exp(−b*(Illuminance + c + e*ERS + M2*(Method == ‘Dark’) + M3*(Method == ‘Clear’))))), fixed = a + b + c + d + e + M2 + M3 ~ 1, random = c ~ 1 | ID).

#### 2.2.3 Advanced model with ERS as an unordered factor

In order to check the linearity assumption of the ERS effect on the logistic function, we ran a final model using the same equation as before but with ERS as an unordered factor. This allows the model to find a coefficient for each value of ERS independently. The model was run both with stats::nls and nlme::nlme, both yielded the same values for the variables of interest. Then, in order to check the effect of ERS, we regressed with a simple linear model the coefficients obtained on ERS.

#### 2.2.4 Model assessment and selection

For both nonlinear least squares (nls) and nonlinear mixed-effects (nlme) models, model fit was evaluated using the log-likelihood, Akaike Information Criterion (AIC), and Bayesian Information Criterion (BIC). AIC and BIC are information-theoretic criteria derived from the log-likelihood and penalized for model complexity (essentially the number of parameters) in different ways. These criteria are useful for model comparison and for the selection of the most parsimonious model with adequate fit.

## 3 Results

The Weber-Fechner law provides valuable insights into the relationship between sensation intensity and stimulus intensity. Specifically, it states that the perceived intensity of a sensation is proportional to the logarithm of stimulus intensity. In this study, we applied this principle by transforming the measured illuminance using decimal logarithms and using transmittance in optical density rather than percentage. The conversion tables that facilitate this transformation can be found in the Supplementary material.

### 3.1 Population

A total of 28 participants (18 women), ranging in age from 18 to 61 years, were recruited for this study. The mean, median and standard deviation of ages of the participants were 36.6, 36.5 and 11.7 years. Eleven participants had emmetropic vision, whereas 17 wore contact lenses. All participants demonstrated a visual acuity of 0.1 Log MAR or better and passed the Ishihara color vision test.

**Figure 1:**
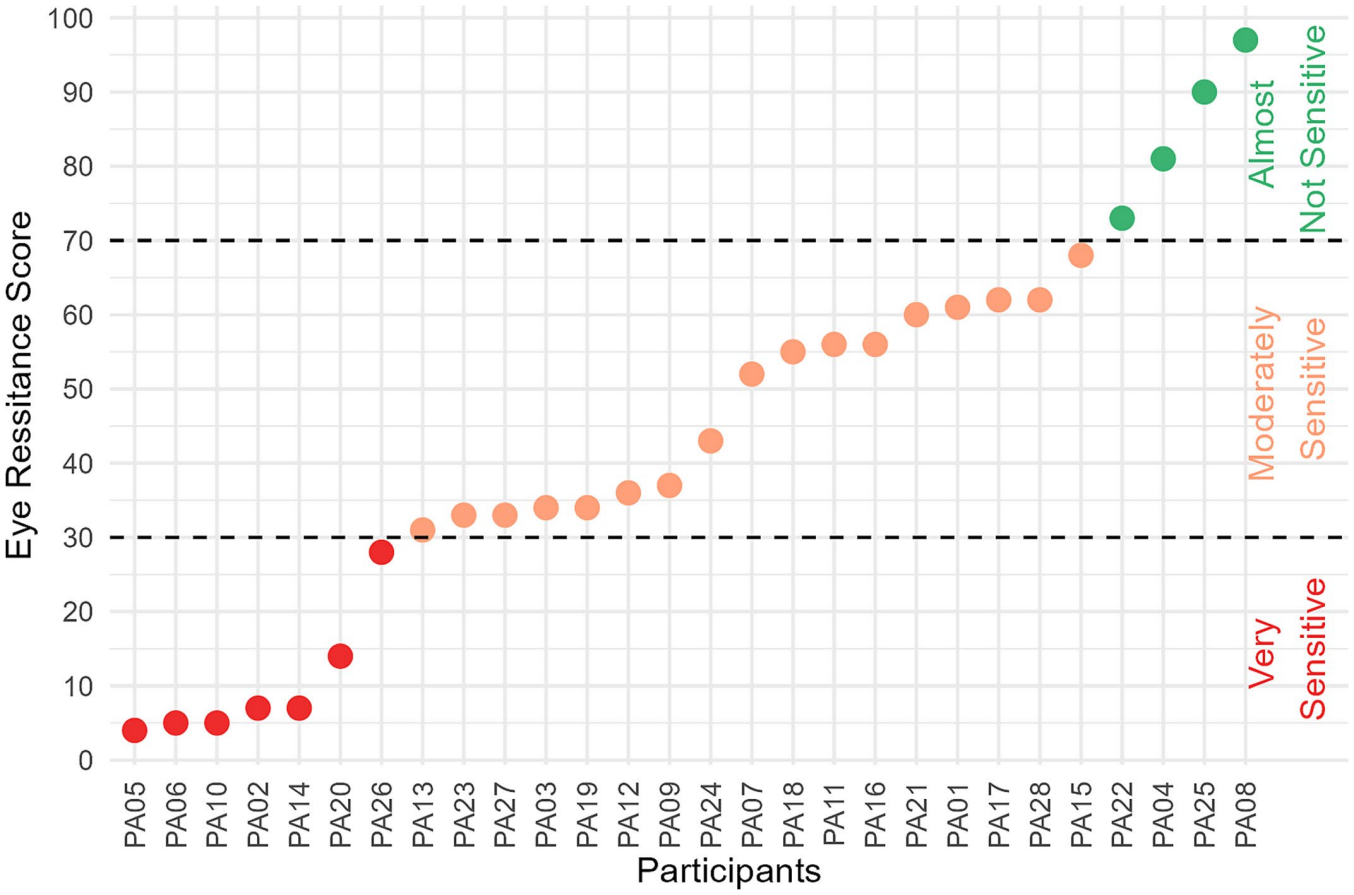
Distribution of eye resistance scores and lumiz segmentation among our study participants with participant ID on the *x*-axis, Eye Resistance Score on the y-axis with 2 horizontal lines representing light sensitivity segmentation limits.

Additionally, Eye Resistance Scores were evenly distributed across the available range, ranging from 0 to 100. The Lumiz segmentation is composed of 3 categories: very sensitive (ERS below 30), moderately sensitive (ERS from 30 to 70) and almost not sensitive (over 70). This approach preserves a broad central group representative of typical sensitivity while maintaining distinct groups at the extremes, which are most relevant for functional interpretation and filter category recommendations. In our study population, Lumiz’s segmentation revealed that 7 participants were very sensitive, 17 were moderately sensitive, and 4 were almost not sensitive (Figure 1).

### 3.2 Light illumination range

**Figure 2:**
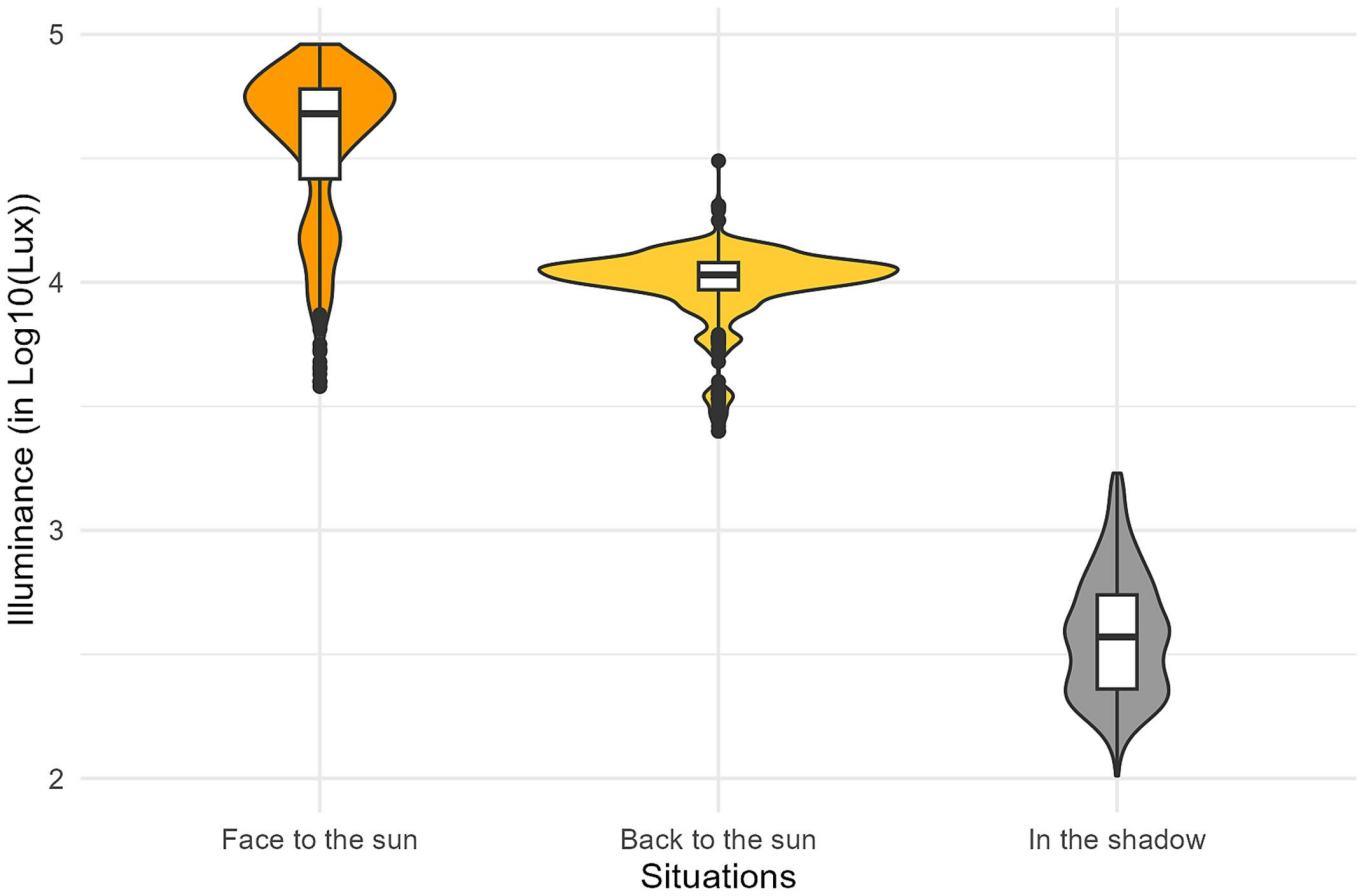
Distribution of illuminance measurements for the 3 light conditions: facing the sun, back to the sun and in the shadow.

Light vertical illuminance measurements, obtained using a calibrated ambient light sensor positioned at the front of the frame of the electrochromic eyeglasses, varied across the different light conditions evaluated (Figure 2). In shaded areas, the illumination is as low as 103 Lux (equivalent to 2.01 Log_10_(Lux)) while, when directly facing the sun, it reaches a peak of 90,200 Lux (equivalent to 4.96 Log_10_(Lux)). The median levels for the specific conditions were:

- Facing the sun: 47,700 Lux (equivalent to 4.68 Log_10_(Lux))
- Back to the sun: 10,700 Lux (equivalent to 4.03 Log_10_(Lux))
- In the Shade: 370 Lux (equivalent to 2.57 Log_10_(Lux)).

The distribution of illuminance facing the sun is bimodal, influenced by weather conditions (sunny vs. overcast). The illuminance values back to the sun have low variance. Illuminance in the shadow is dispersed but follows a unimodal pattern. Two situations (facing the sun and back to the sun) have outliers. Lower outliers correspond to highly overcast weather conditions. Upper outliers in the back to the sun condition may have been caused by specular reflections of sunlight on a nearby window. Overall, these conditions exhibit distinct variations in illumination.

### 3.3 Modeling of transmittance setting

#### 3.3.1 Initial model using only illuminance

Here, we model the association between transmission settings recorded with three different methods and illuminance recorded in three real outdoor lighting conditions. The transmittance of an ophthalmic filter ranges from 3 to 100% (optical density from 0 to 1.52). In our setting, the minimum and maximum transmittances were, respectively, 1.3 OD (i.e., 5%) and 0.1 OD (i.e., 80%). We observed some saturation: the lens darkness was measured as insufficient in some cases (the observer could not set the lens dark enough to be comfortable in the light condition), whereas the clearest filter could be selected for a large range of illuminations from no light up to an initial illumination level where light produces the first sensation of discomfort. This led us to model transmittance as a logistic function of illuminance, which allows us to describe the selection of the clearest filter for a range of low illumination, and similarly, to describe the selection of the darkest filter for a range of high illumination when darker filters would be selected (but not available).

**Figure 3:**
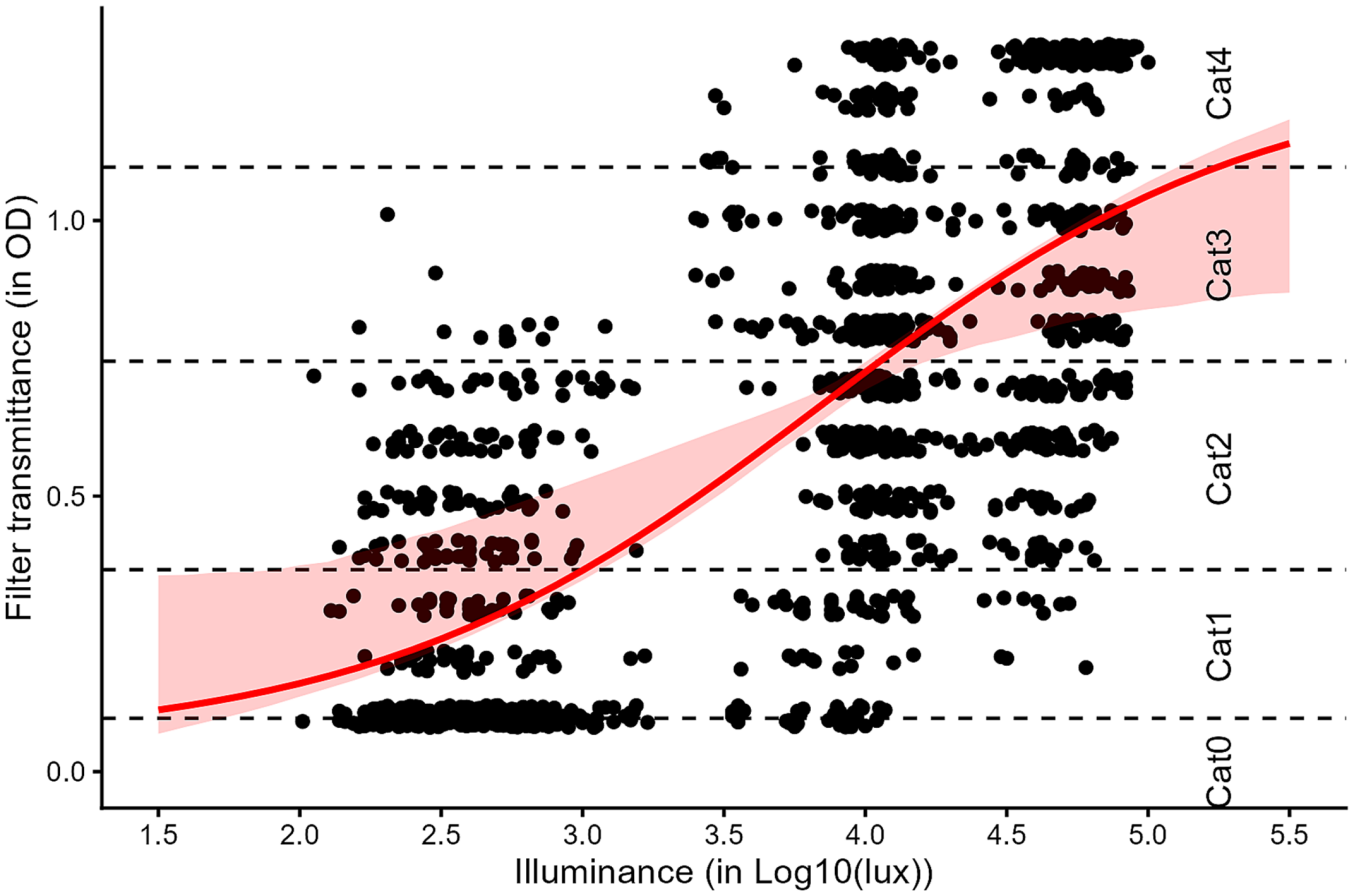
Model filter transmittance as a function of illuminance using a logistic function. The shaded red area corresponds to the bootstrapped 95% confidence interval. Dashed line represents filter category (from class 0 to class 4). A little vertical jitter was added for the sake of clarity in this figure and all following ones.

The model of transmittance as a logistic function of illuminance (see Materials and Methods for a description and Supplementary material for detailed results) was significantly better than the null model (consisting only of an intercept) (*F_3,1,526_ = 523.2, p < 0.001*). This generic model demonstrates (Figure 3) the need for filter category 2 (light transmittance between 43 and 18%) at 100 Lux up to category 3 (light transmittance between 18% and 8%) at 10,000 Lux. So far, this simply tells us that illuminance impacts the optimal transmittance setting.

#### 3.3.2 Advanced model using illuminance, method and eye resistance score

In the study population, we observed a wide range of light sensitivities as recorded by the Lumiz 100™ (ERS from 4 to 97). We hypothesized that the transmittance settings would differ according to the light sensitivity. Moreover, we considered the method (Clearest-to-Dark, Darkest-to-Clear, Manual) may also affect the transmittance settings. We ran models including the fixed effects method and/or ERS as horizontal shifts of the logistic and subject ID as random effects (models 2, 3 and 4, see Materials and Methods). These models were significantly better than the initial model using only illuminance (see Table 1): the AIC and BIC of the simple model (without random effects altogether) with df = 5 were 469.6 and 496.3 while for the complex model with df = 9 they were −405.1 and −357.1. Compared to the model with illuminance only but still with subject ID as random effects, the likelihood ratio test statistic was 111.5 (successive tests yielding 27.4 + 56.6 + 27.5) indicating that including Method and ERS in the model make it vastly superior to the model with illuminance only.

**Table 1:** Synoptic summary of the various models. Lux stands for illuminance, df for degrees-of-freedom, AIC and BIC for Akaike and Bayes Information Criteria, LogLik for log-likelihood and L.ratio for likelihood ratio.

Parameters	Model	df	AIC	BIC	LogLik	Test	L.ratio	*p*-value
Lux (nls without random effect)	0	5	469.6	496.3	−229.8			
Lux (with RE)	1	6	−299.7	−267.7	155.8			
Lux + ERS (with RE)	2	7	−325.1	−287.7	169.5	1 vs. 2	27.4	1.6 10^−7^
Lux + Method (with RE)	3	8	−379.6	−337.0	197.8	2 vs. 3	56.6	5.4 10^−14^
Lux + Method + ERS (with RE)	4	9	−405.1	−357.1	211.6	3 vs. 4	27.5	1.6 10^−7^

Model 0 only features illuminance without random effects, while all following ones integrate participant ID as random effect (RE).

In the following two sections, we explore the results of Model 4 in terms of Method and ERS.

##### 3.3.2.1 Effect of method

**Figure 4:**
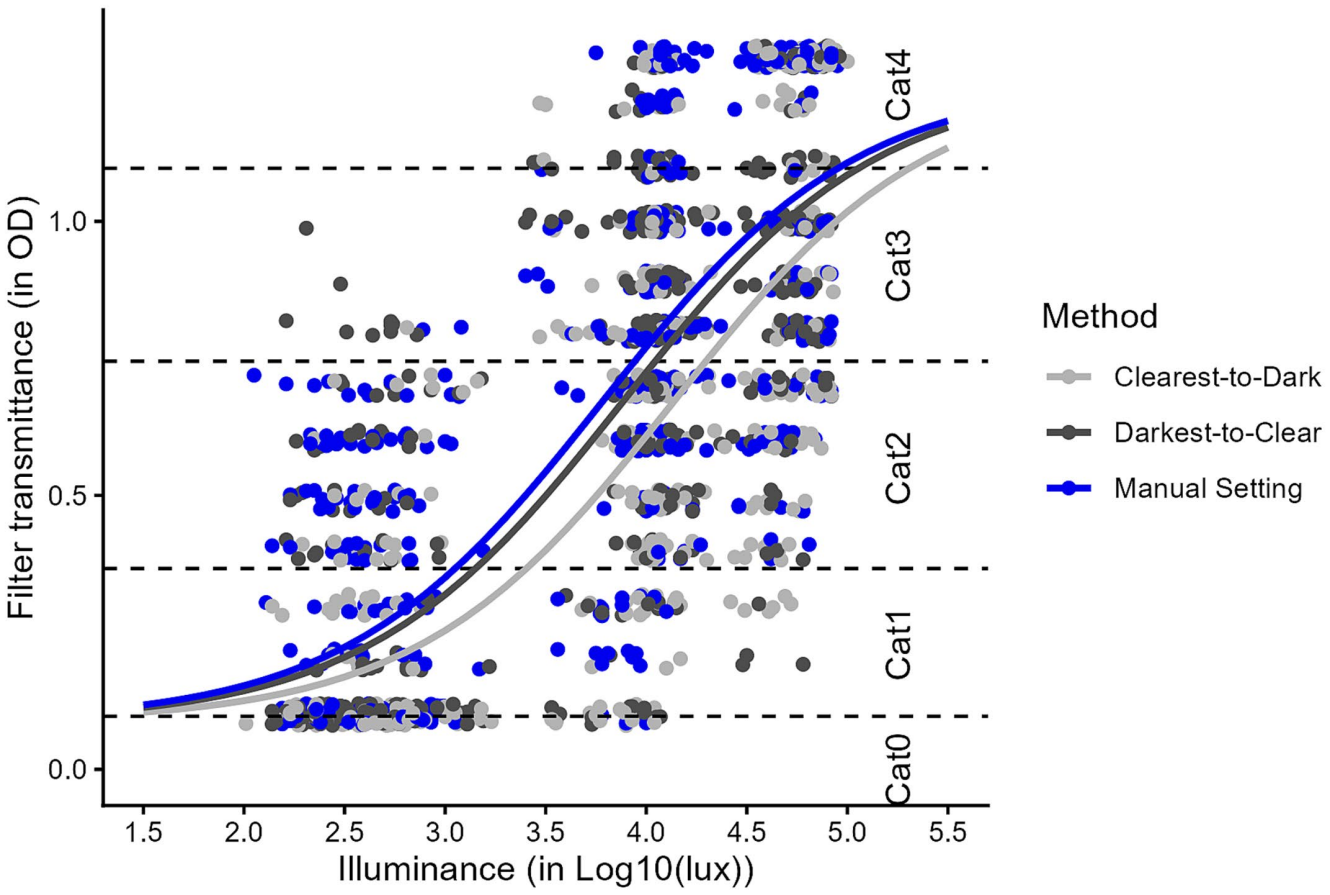
Model of filter transmittance (in OD) on the y-axis as a function of illuminance and method (for ERS = 50) with illuminance (in log_10_(Lux)) on the x-axis.

The choice of method for determining the transmittance setting under varying light conditions affects the measurement outcomes (Figure 4). Specifically, the Clearest-to-Dark method yields clearer transmittance level settings compared to both the Darkest-to-Clear and Manual Setting methods. While the difference is minimal under low illumination, it increases up to 0.16 OD for illumination levels exceeding 1,000 Lux (3 Log_10_(Lux)). Notably, the difference between the Darkest-to-Clear and Manual methods was negligible (0.05 OD). This effect is significant as per Table 1.

##### 3.3.2.2 Effect of ERS

**Figure 5:**
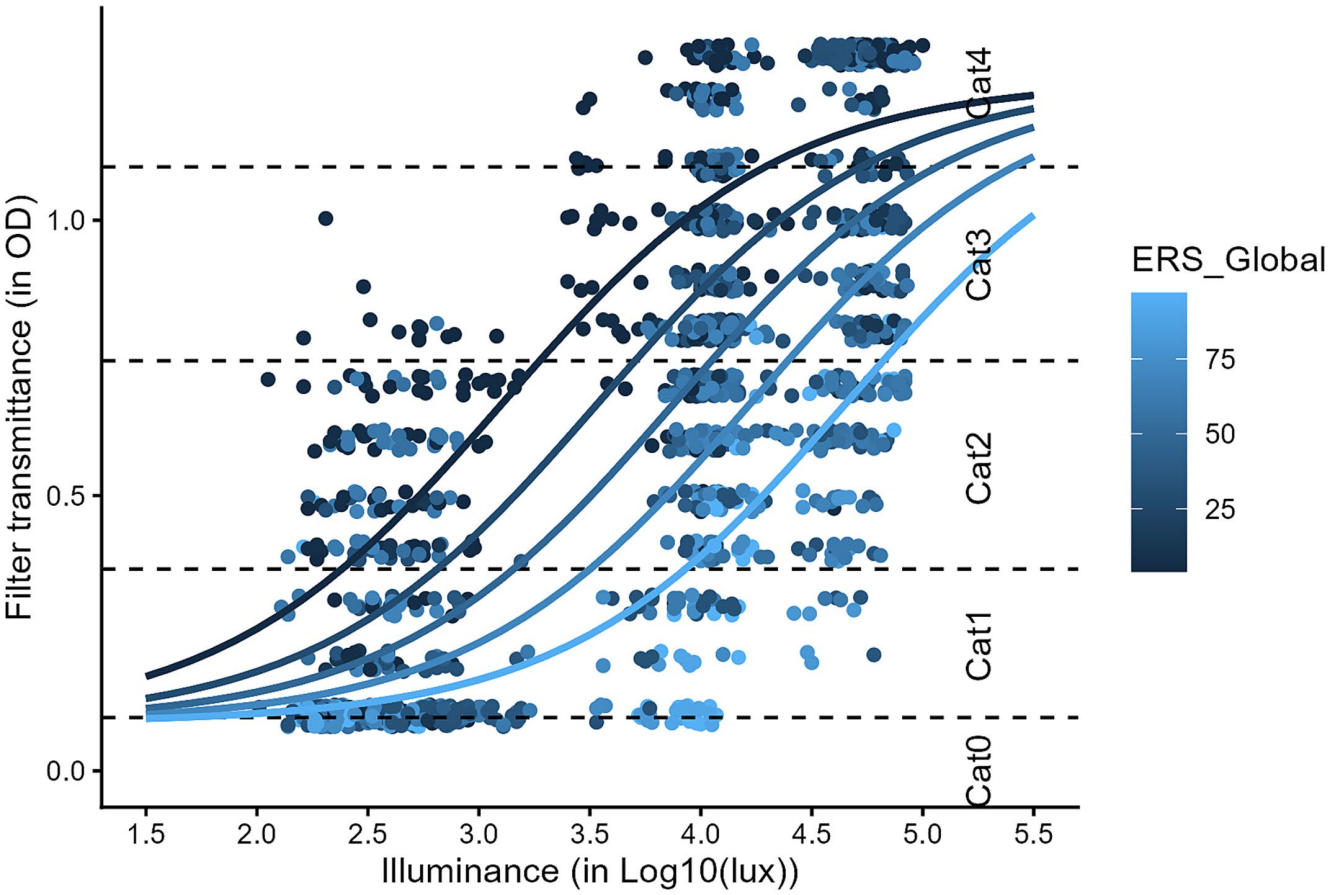
Model of filter transmittance (in OD) in y-axis as a function of illuminance (in log_10_(Lux)) in x-axis and Eye Resistance Score (for the Manual Setting Method).

The advanced model 4 shows that the Eye Resistance Score affects the optimal transmittance settings (Figure 5). Each additional point of ERS shifts the logistic curve 0.0172 points to the right. Less sensitive participants would only require a category 2 filter (light transmittance greater than 18% and up to 43%) for illumination exceeding 10,000 Lux (equivalent to 4log_10_(Lux)). Conversely, more sensitive participants would need additional category levels from the category 2 filter to the category 4 filter (light transmittance greater than 3% and up to 8%). For low illumination, corresponding to shadow situations between 100 and 1,000 Lux (equivalent to 2 to 3 log_10_(Lux)), the settings correspond to a category 2 filter. For intermediate illumination, representative of situations with the sun at one’s back, ranging from 1,000 to 10,000 Lux (equivalent to 3 to 4log_10_(Lux)), the settings correspond to a category 3 filter (light transmittance greater than 8% and up to 18%). For the highest illumination, indicative of facing direct sunlight over 10,000 Lux (equivalent to 4log_10_(Lux)), the settings correspond to a category 4 filter.

#### 3.3.3 Advanced model with ERS as an unordered factor

**Figure 6:**
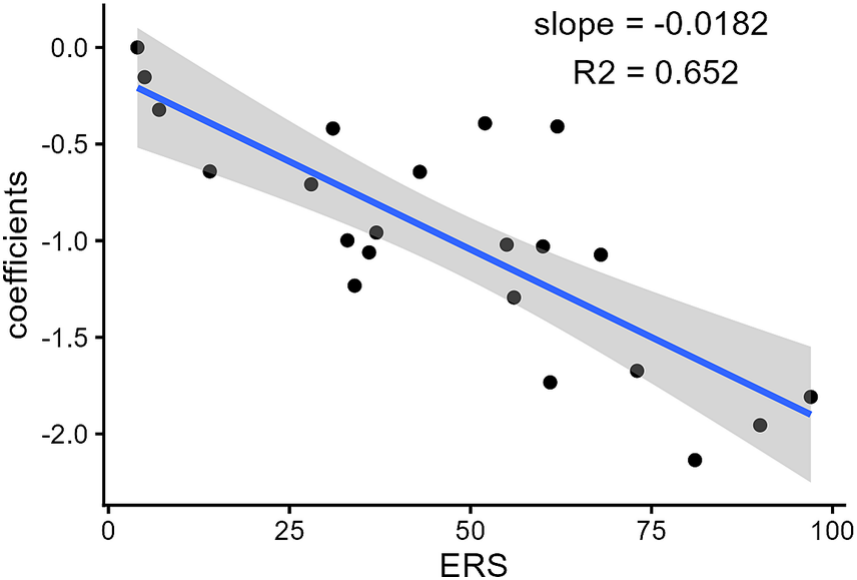
Coefficients for Eye Resistance Score on the y-axis of the advanced model as a function of Eye Resistance Score on the x-axis. The link between computed coefficient and Eye Resistance Score has a correlation of *r*^2^ = 0.652 and a slope of −0.0182.

This last model evaluates the linearity assumption of the effect of ERS on the logistic function (Figure 6). To assess the validity of this assumption, we ran a model where ERS is not treated as a continuous variable, but rather as an unordered factor, thereby giving it complete freedom, rather than ordered, not to enforce any trend between the ERS and the coefficient estimates. In this model, a horizontal shift is estimated for each value of ERS independently. We then observed the relationship between ERS and these shifts (see Figure 6), and found a linear link between them with fairly large goodness-of-fit (*R*^2^ = 0.652) and with a slope that is very close to the one obtained in the previous model (−0.0182 vs. − 0.0172, see Supplementary material 8.2, model 4) suggesting that the linearity assumption is warranted.

## 4 Discussion

Visual perception is intrinsically influenced by the luminance of the observed environment. In this study, due to feasibility constraints, we measured vertical illuminance using a calibrated ambient light sensor as a proxy for light environment. While this method provided a practical and consistent approach for data collection, it represents an approximation, as illuminance does not fully account for the spatial and directional characteristics of luminance. Moreover, a real-time assessment of what is actually perceived during visual tasks would require the combination of luminance maps with eye-tracking technology. This would allow for a fine-grained evaluation of the specific areas being observed during adjustment or interaction. Such an approach was beyond the scope of the present study but could be considered in future research to enhance the precision of perceptual analysis. Nevertheless, the strong and systematic relationship between measured illuminance and selected filter transmittance observed across conditions suggests that vertical illuminance constitutes a relevant proxy for the overall visual load experienced during everyday outdoor activities. The ability of the logistic model to robustly capture transmittance selection supports the ecological validity of this simplified metric in real-world contexts.

The study was conducted during the summer in a temperate zone within an urban environment surrounded by buildings. The observed lighting conditions were typical of a summer day in Paris, France with both sunny and overcast weather. At a comparable latitude, but in an unobstructed environment, the illuminance levels would be higher. Furthermore, in tropical and equatorial climates, the illuminance notably increases. The lighting conditions observed in this study under direct sunlight were high, but not as extreme as those encountered in snowy environments or at the beach, with significant ground reflections. The findings of this study were specific to the lighting conditions during the test. Further investigations would be relevant for brighter environments to explore their effects on transmittance settings. However, these conditions remain representative of the daily outdoor exposures encountered by urban populations at mid-latitudes. As such, the present results are highly relevant for common activities such as commuting, walking, and leisure, which account for a substantial proportion of sunglasses use in everyday life.

Similarly, the available transmittance of the electrochromic glasses ranges from 80 to 5%. Some measurements suggested an insufficient range: darker filters, and occasionally clearer ones, could have been chosen. A wider transmittance range would not significantly impact the clear filter zone, but it would likely affect the transmittance settings for darker filters. Importantly, the occurrence of saturation at both ends of the available transmittance range is informative. It highlights illuminance levels where widely available category 3 filters may be either more than sufficient or insufficient for certain individuals, particularly among the most light-sensitive participants. Therefore, to better accommodate interindividual variability under high illuminance conditions, a wider range of filters should be proposed to customers.

In retail stores, the majority of available sunglasses fall into category 3 darkness (light transmittance between 18% and 8%). The participants’ awareness of the darkness of typical sunglasses likely influenced their settings. To prevent the direct observation of transmittance level when removing typical filters, we used adaptative electrochromic glasses and we implemented three methods to evaluate optimal settings. Additionally, discomfort glare is affected by visual adaptation, a process that involves dynamic changes in retinal sensitivity and pupil diameter in response to ambient luminance, which occur over characteristic time scales ranging from seconds to several minutes. Therefore, we introduced both the Clearest-to-Dark and Darkest-to-Clear methods, which simulate real-world scenarios involving changes in illuminance. The Manual Setting method imposes fewer constraints and allows time for light adaptation. In the study results, the difference between the Darkest-to-Clear and Manual methods is small. Interestingly, the observed shift between the Clearest-to-Dark Method and Darkest-to-Clear method may be attributed to adaptation of the visual system. In glare situations (filter too clear), the visual system attempts to adapt to limit discomfort. In this way, transmittance is set at lower darkness compared to a method in which the visual system does not need to adapt to a high illuminance. However, the difference in transmittance settings between different methods is moderate (up to 0.15 Log_10_(Lux)). This demonstrates that the adaptation of the visual system is limited within the time windows of interest and lower than the filter category range.

This study highlights significant differences in optimal transmittance settings based on light sensitivity, as measured by the Lumiz 100™ tool and the Eye Resistance Score. Among the healthy population of 28 participants, less sensitive individuals may require a category 2 filter (light transmittance between 43 and 18%) in very bright conditions, while the most sensitive would benefit from a category 2 filter in shadowed areas and up to a category 4 filter (light transmittance between 8 and 3%) in direct sunlight. Even though the category 4 filters are prohibited for driving due to reduced visibility in lower light conditions experimented while driving such as tunnel or indoor parking, it would benefit the very sensitive population in their other everyday-life outdoor activities. Although most sunglasses fall into category 3 (light transmittance between 18 and 8%), personalized recommendations for filter darkness, considering an individual’s typical light environment, activities (such as driving, commuting or outdoor sports), and UV protection are advantageous. However, for specific activities with high illuminance levels in snowy or seaside environments, the moderately sensitive population could benefit from a filter with lower light transmittance than a category 3 filter. As explained before, the light conditions for this study were typical of our location. But for a population located in an environment with higher illuminance such as a non-obstructed environment or a location with tropical or equatorial climates, we could imagine that not only the very sensitive population, but also a part of the moderately sensitive would benefit from a category 4 filter in their everyday-life outdoor activities.

It should be noted that the present study specifically assessed discomfort glare and subjective visual comfort, without directly measuring visual performance metrics such as contrast sensitivity, reaction time, or object detection. While darker filters generally improve comfort under high illumination, excessively low transmittance may affect certain visual tasks depending on context. Therefore, the filter preferences reported here should be interpreted as comfort-based recommendations rather than comprehensive prescriptions for all visual activities.

This study opens up a new field of exploration regarding light sensitivity and the benefits of filters in reducing discomfort glare, measuring filter needs in outdoor light environment conditions for the first time. Previous studies have hypothesized various factors, including fatigue level, food ingestion, physical or emotional state, and prior light exposure (Kent et al., 2016; Pierson et al., 2018; Van Den Wymelenberg, 2012). Different lighting conditions across various environments and latitudes would also affect filter needs. Continued research in this area is valuable for advancing our understanding of light sensitivity and filter glare protection.

## 5 Conclusion

This study investigated discomfort glare filter preferences in real outdoor environments using electrochromic lenses and psychometric adjustment methods, with explicit consideration of individual light sensitivity. By measuring transmittance settings across a wide range of illuminance levels and outdoor configurations, we showed that filter selection cannot be explained by ambient light intensity alone.

Incorporating Eye Resistance Score, assessed with the Lumiz 100™ device, significantly improved the prediction of preferred transmittance, revealing substantial interindividual variability. While less light-sensitive participants generally selected category 2 filters under high illumination, more sensitive participants required substantially darker filters, corresponding to category 4, to achieve visual comfort in direct sunlight. Note that methodological effects related to adjustment procedures were also observed.

Overall, these findings support personalized approaches to filter recommendations that jointly consider environmental illuminance and individual sensitivity, with potential benefits for visual comfort and safety in everyday outdoor activities. Further research in a broader range of environments and lighting conditions will help refine these recommendations.

## Data Availability

The data supporting the conclusion of this article is not publicly available due to ethical considerations in accordance with applicable data protection regulations for human subjects research. However, requests to access the datasets may be asked to the corresponding author and will be subject to case-by-case review.
