# Supplementary material for: Improving filter recommendations: the role of individual light sensitivity in ecological conditions
Source: Front Psychol. 2026 Jun 30;17:1755789. doi: 10.3389/fpsyg.2026.1755789 (PMC13365335; doi:10.3389/fpsyg.2026.1755789)
Supplement: Supplementary file 1 [file Supplementary_File_1.pdf]

Improving Filter Recommendations: The Role of Individual Light Sensitivity in Ecological ConditionsErreur ! Source du renvoi introuvable.

Supplementary

1 Log correspondence (Transmittance & Illumination)

Illumination (Lux)	Illumination (Log_{10}(Lux))	Transmittance (%)	Optical Density (Log_{10}(1/Tr))
**100 000**	5,00	100%	0,00
**80 000**	4,90	80%	0,10
**60 000**	4,78	63%	0,20
**40 000**	4,60	50%	0,30
**20 000**	4,30	40%	0,40
**10 000**	4,00	32%	0,49
**8 000**	3,90	25%	0,60
**6 000**	3,78	20%	0,70
**4 000**	3,60	16%	0,80
**2 000**	3,30	13%	0,89
**1 000**	3,00	10%	1,00
**800**	2,90	8%	1,10
**600**	2,78	6%	1,22
**400**	2,60	5%	1,30
**200**	2,30	4%	1,40

2 Coefficients from models

Coefficients for the initial model 0:

	Estimate	Std. Error	t-value	p-value
a	1.218	0.298	4.08	<0.0001
b	1.273	0.422	3.06	0.002
c	-3.836	0.131	-29.34	<0.0001
d	0.053	0.133	0.42	0.68

Coefficients for the initial model 1:

	Estimate	Std. Error	t-value	p-value
a	1.166	0.058	19.98	<0.0001
b	1.587	0.134	11.88	<0.0001
c	-3.964	0.115	-34.51	<0.0001
d	0.097	0.025	3.88	<0.0001

Coefficients for the advanced model 2 (ERS):

	Estimate	Std. Error	t-value	p-value
a	1.160	0.057	20.21	<0.0001
b	1.596	0.134	11.96	<0.0001
c	-3.214	0.136	-23.60	<0.0001
d	0.098	0.025	3.95	<0.0001
e	-0.017	0.003	-6.71	<0.0001

Coefficients for the advanced model 3 (Method):

	Estimate	Std. Error	t-value	p-value
a	1.170	0.054	21.58	<0.0001
b	1.577	0.124	12.72	<0.0001
c	-3.783	0.118	-32.14	<0.0001
d	0.087	0.024	3.57	0.0004
M2	-0.354	0.040	-8.89	<0.0001
M3	-0.102	0.040	-2.57	0.0104

Coefficients for the advanced model 4 (ERS + Method):

	Estimate	Std. Error	t-value	p-value
a	1.165	0.053	21.79	<0.0001
b	1.586	0.124	12.78	<0.0001
c	-3.025	0.139	-21.71	<0.0001
d	0.087	0.024	3.63	0.0003
e	-0.017	0.003	-6.73	<0.0001
M2	-0.355	0.040	-8.90	<0.0001
M3	-0.102	0.034	-2.56	0.0105
